# Make it even better

**DOI:** 10.1172/JCI158458

**Published:** 2022-03-01

**Authors:** Elizabeth M. McNally

“Just because something works doesn’t mean it cannot be improved.”

—Shuri, *Black Panther* (2018), Marvel Comics

As I assume the editorship of the *Journal of Clinical Investigation*, I look to those who came before me to learn from their experience and to chart the course as we steer forward. The Northwestern team is honored to follow in the footsteps of Johns Hopkins and the NIH, Duke and the University of North Carolina, the University of Pennsylvania, Columbia University, and many prior esteemed boards. Dr. Rexford S. Ahima, the outgoing Editor in Chief, skillfully guided a midterm leadership transition from Dr. Gordon F. Tomaselli, and during this time, Dr. Kathleen L. Collins expertly navigated *JCI Insight* to full editorial autonomy at the University of Michigan. The journal family of *JCI* and *JCI Insight* carries a strong reputation of the highest-quality science in a broad range of disease-relevant biology. In a nutshell, the *JCI* works. Yet the *JCI* can be improved, just as the brilliant scientist Shuri from Marvel’s *Black Panther* continued to improve the wonders of Wakanda.

The *JCI* was born in 1924, a time when biomedical science was embryonic. The first papers published in the *JCI* described blood gas content during pneumonia and the specific gravity of urine (1, 2). Early in its course, the *JCI* published experiments using animal models to describe key pathophysiology across a spectrum of systems. In 1925, a paper on the effect of pyloric obstruction in rabbits listed its authors as James L. Gamble and Monroe A. McIver “with assistance of Pauline Marsh,” making Ms. Marsh the first woman to publish in the *JCI* (3). In that same year, the *JCI* published its first proceedings of an annual meeting of the American Society for Clinical Investigation (ASCI; its 17th), including abstracts describing how to culture vaccine virus and features of recurrent hay fever, reinforcing the consistent theme of the scientific inquiry of human disease (4).

Moving through the near-century-long history of the Journal, the *JCI* published more work that relied on experimental nonhuman animal models, to the degree that some questioned whether the *JCI* was still publishing human clinical investigation. The use of genetically engineered models revolutionized our molecular understanding of countless physiological processes, but at times, these findings may have been lost on clinicians and may not have always accurately reflected human disease. I am delighted to report there is now increasing presence of human investigation in the *JCI*, and this is an area we hope to expand even more because the time is right. The availability of human clinical data has never been greater, and new methods and tools to explore human pathophysiology are being developed each day.

The *JCI* is broadly interested in human investigation that defines disease processes, from rare to common. With expanded ability to phenotype and genetically profile, rare-disease or even “*n* of one” studies can uncover entirely new and relevant pathways of disease. At the other end of the spectrum, there is great science coming from large data sets containing human health information — from electronic health records to deidentified “omic” data sets. “Big data,” parsed by human or artificial intelligence, can frame exceedingly good questions. The *JCI* seeks papers making novel observations and/or testing hypotheses derived from use of human data, cell, or animal modeling. The Journal will continue to publish papers using translationally relevant, genetically engineered models, since these remain a rigorous means of demonstrating fundamental in vivo mechanisms. At the core of the *JCI* is the mission to explain human disease phenomena in a rigorous and reliable manner. Testing of hypotheses may utilize genetic or pharmacological methods and, in so doing, establish therapeutic pathways, and the model systems can be human cells, organoids, or nonhuman cells or organisms. As scientists, we are driven to understand how things work. As disease biologists, we use pathology to define mechanisms and experimentally intervene to correct the underlying defects. Now at the dawn of therapeutic gene editing, a method we have had for less than 10 years, so many things are possible for the future of medicine. We want to see these papers in the *JCI*.

Scientists and physician-scientists come in all shapes and sizes, from a range of cultural and educational exposures, and this diversity helps us be better scientists. Photographs documenting the ASCI’s early history are notable for their homogeneity and often conveyed the message that only those with a specific social and educational upbringing were welcome. So many creative minds did not have the opportunity to become scientists. Although much work remains in order to improve diversity in science, many inroads have been made in the ensuing decades, and we now recognize great science can be found in all corners of the world. Moreover, reflecting the changing demographics in the United States, the younger generation of scientists is more likely to hail from diverse socioeconomic, cultural, racial, and ethnic backgrounds. This diversity brings new points of view and new solutions to challenging biomedical problems. At the same time, the experienced investigator often has new thoughts and approaches that derive from a lifetime of experience. At the *JCI*, the science will be fairly weighed, no matter who submits it and no matter where they come from.

As incoming Editor in Chief, an author, and a mentor, I am sensitive to how long manuscripts remain in the submission, revision, and re-revision process. We probably all know authors who endured 2 or more years of work *after* a manuscript’s initial submission to have their findings accepted for publication. Two years is too long. For investigators at all stages and especially trainees, important discoveries should appear in the peer-reviewed public domain more quickly. I hope to shorten the review/revision process by clarifying instructions to authors and making earlier decisions about whether a manuscript fits the mission of the *JCI*. I would like to avoid overburdening reviewers while ensuring that the published data sit at the right intersection of novelty, impact, and accuracy. In 2022, we plan to update guidance to reviewers about our expectations.

We also plan to better measure the impact of the findings published in the *JCI* and expand the impact of the *JCI*’s featured science. The *JCI* has been an “open-access” journal since 1996, when the Journal first appeared online, publishing all research outside a paywall and available to anyone, whether that is your colleague, competitor, a potential investor, your patients, or even your parents. In 2022, the *JCI* became Gold Open Access, so that all content is available without charge to the user or their institution. We seek to publicize the work in the *JCI* more effectively so that your work is better seen, understood, and appreciated. Social media platforms promote scientific dissemination, and publicly available links to open-access material brings greater visibility. The global pandemic reduced conventional face-to-face scientific exchange, yet it markedly expanded electronic information exchange through preprint servers and social media. Reputable journals such as the *JCI* can serve as an endorsement of impact and veracity, which can magnify the impression and influence of the findings.

The *JCI*’s first issue coincided with the opening of Soldier Field, the home of the Chicago Bears. In 2024, the *JCI* will mark its 100th anniversary, and here at Northwestern, we will celebrate this century of biomedical science and human investigation. Ultimately, the *JCI* represents the American Society for Clinical investigation, an academically directed enterprise. We invite you to be part of the process by reading the *JCI*, reviewing submissions, and choosing the *JCI* as home for your best work.

Our Northwestern-based editorial team, augmented with colleagues from the University of Chicago, are the stewards of the Journal for the next 5 years. Collectively, our Editorial Board has remarkably broad expertise, drawing from strengths in cardiology, oncology, vascular biology, transplantation, the microbiome, immunology, genomics, human genetics, population science, computational biology, artificial intelligence, gene therapy, clinical trials, and more. Look to our website for more details about the Deputy Editors and Associate Editors and their expertise. We plan to highlight exciting work at the interface of scientific disciplines through publication of Editor’s notes, and we expect to diversify authors and reviewers through outreach. The *JCI* has a robust group of consulting editors, who serve as regular reviewers, and we envision asking some of these individuals to serve as guest editors in the future to further expand our editorial domain knowledge. We acknowledge the *JCI*’s strong reputation, we know we can do even better, and we hope its readership, authors, and reviewers will help us achieve this goal.

## Version 1 03/01/2022

Electronic publication
